# Genomic analysis and prediction of genomic values for distichiasis in Staffordshire bull terriers

**DOI:** 10.1186/s40575-023-00132-1

**Published:** 2023-07-24

**Authors:** Dina Jørgensen, Ernst-Otto Ropstad, Theodorus Meuwissen, Frode Lingaas

**Affiliations:** 1 Medical Genetics Unit, Faculty of Veterinary Medicine, Norwegian University of Life Sciences, P.O. box 5003, 1432 Ås, Norway; 2 Evidensia Oslo Dyresykehus, Ensjøveien 14, 0655 Oslo, Norway; 3 Department of Animal and Aquacultural Sciences, Faculty of Biosciences, Norwegian University of Life Sciences, P.O. box 5003, 1432 Ås, Norway

**Keywords:** Canine, Genomic prediction, Distichiasis, GWAS, Staffordshire bull terrier

## Abstract

**Background:**

Distichiasis is a condition characterized by aberrant hairs along the eyelid margins. The symptoms are usually mild but can lead to ulcerations and lesions of the cornea in severe cases. It is the most frequently noted ocular disorder in Norwegian Staffordshire bull terriers (SBT), with a prevalence above 18% in the adult population. A complex inheritance is assumed, but there is sparse knowledge about the genetic background of distichiasis in dogs. We have performed a genome-wide association study of distichiasis in SBT and used genomic data in an attempt to predict genomic values for the disorder.

**Results:**

We identified four genetic regions on CFA1, CFA18, CFA32 and CFA34 using a mixed linear model association analysis and a Bayesian mixed model analysis. Genomic values were predicted using GBLUP and a Bayesian approach, BayesR. The genomic prediction showed that the 1/4 of dogs with predicted values most likely to acquire distichiasis had a 3.9 -4.0 times higher risk of developing distichiasis compared to the quarter (1/4) of dogs least likely to acquire the disease. There was no significant difference between the two methods used.

**Conclusion:**

Four genomic regions associated with distichiasis were discovered in the association analysis, suggesting that distichiasis in SBT is a complex trait involving numerous loci. The four associated regions need to be confirmed in an independent sample. We also used all 95 K SNPs for genomic prediction and showed that genomic prediction can be a helpful tool in selective breeding schemes at breed level aiming at reducing the prevalence of distichiasis in SBTs in the future, even if the predictive value of single dogs may be low.

**Supplementary Information:**

The online version contains supplementary material available at 10.1186/s40575-023-00132-1.

## Abstract (plain language summary)

Distichiasis is a condition where abnormal hairs grow along the margin of the eyelids. It's common in Staffordshire bull terriers and can cause eye problems of variable severity. The abnormal eye hairs can be found during an eye inspection performed by a veterinarian.

We performed a genome-wide association analysis and identified four genomic areas associated with the condition. But more genes may be involved in causing the disease.

We have used genomic data to predict genomic values. Genomic values can be used to predict the total load of disease-associated alleles. Genomic prediction would therefore be helpful at the breed level, similar to pedigree-based breeding values, to reduce the prevalence of dogs with distichiasis, even if the low accuracy to predict phenotypes in individual dogs may be a challenge. More research is needed to confirm these findings and see if genomic prediction could be a helpful tool within dog breeding in the future.

**Supplementary Information:**

The online version contains supplementary material available at 10.1186/s40575-023-00132-1.

## Background

Distichiasis is a condition with abnormal growth of eye hairs along the margins of the eyelid. The aberrant hairs arise from ectopic hair follicles near the meibomian glands and emerge through the excretory duct opening of the sebaceous glands [1, 2]. In most cases, the symptoms caused by distichiasis are mild. Eye irritation with increased lacrimation and conjunctivitis can be seen. In severe cases, the aberrant eye hair can lead to lesions of the cornea with ulcerations and keratitis [3].

Distichiasis is common in dogs [3, 4] and the most frequently noted ocular disorder in Staffordshire bull terriers (SBT) in Norway [5]. In a previous study, we found a prevalence of 18.72% among Norwegian SBTs examined after one year of age, and the heritability was estimated to be moderate to high [6]. The same level of heritability has been seen in the dog breeds; havanais [7], elo [8] and cocker spaniels [9]. A simple Mendelian inheritance was excluded in a segregation analysis in elos, however, the exact mode of inheritance was not defined [10]. A complex mode of inheritance involving multiple genes is assumed. Thus far, little is known about the genetic background of distichiasis in dogs.

Distichiasis is less common in other species than the dog but has been described in cats [11], ferrets [12], cattle [13, 14], and horses [15]. In Friesian horses, Hisey et al. found a 16 kb deletion in an intergenic region on equine chromosome 13 associated with distichiasis, and a dominant inheritance with incomplete penetrance is assumed [15]. In cattle, distichiasis has been associated with the autosomal dominant *Polled* locus on the bovine chromosome 1 [14]. In humans, distichiasis has been associated with an autosomal dominant mutation in the region of the *FOXC2* gene, both alone and as a part of a syndrome with lymphedema [16–18]. Other rare conditions in humans seen in combination with distichiasis are facial dermal dysplasia caused by a frameshift mutation in *TWIST2* [19] and Blepharocheilodontic syndrome linked to mutations in *CTNND1* and *CDH1* encoding proteins in the cadherin–catenin complex [20]. So far, no genes or genetic regions have been found to be associated with distichiasis in dogs.

We were interested in using distichiasis as a model for canine genomic prediction, by estimating the joint effect of all genomic markers to predict a phenotype. Genomic predictions have had great success in livestock breeding [21]. Until now, genomic predictions have received little attention in dog breeding. There have been a few studies using genomic SNP data to predict disorders such as canine hip dysplasia [22, 23], cranial cruciate ligament rupture [24], and kidney disease [25]. Thorsrud et all. compared genomic best linear unbiased prediction (GBLUP) with four different machine learning techniques to predict distichiasis, mandibular distocclusion and acral lick dermatitis in a guide dog population consisting of German shepards, golden retrievers, Labrador retrievers, and Labrador and golden retriever mixes [26].

In the present study, we aim to investigate the genetic background of distichiasis in SBTs through a genome-wide association analysis (GWAS). We have compared two approaches for genomic prediction: GBLUP and BayesR and have used the results to investigate the potential value of using genomic data to predict genomic values for the disorder in SBTs.

## Results

### Genome-wide association study

**Fig. 1:**
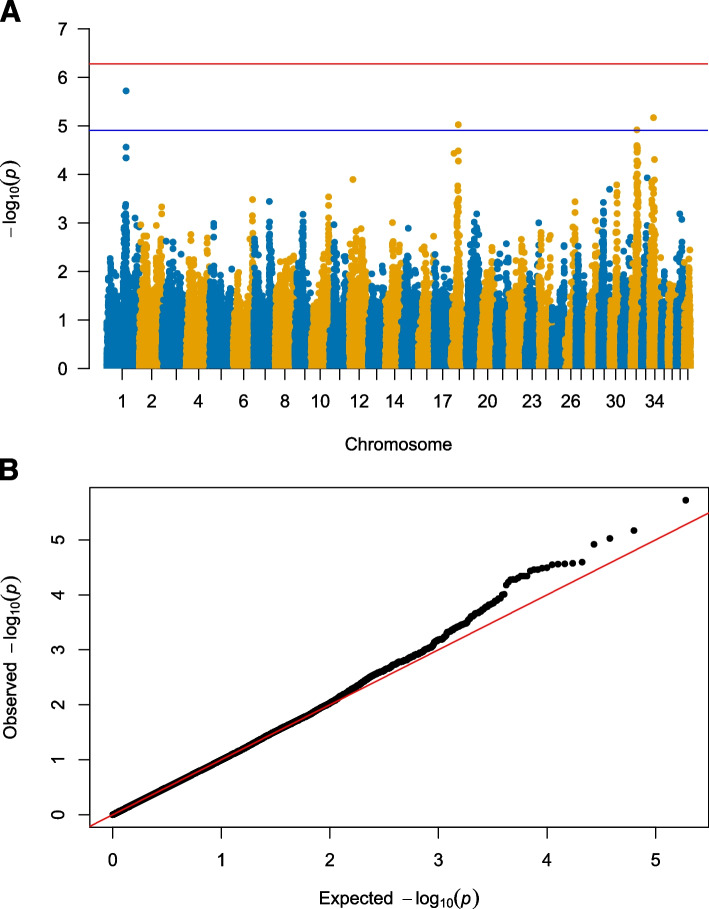
Mixed linear model association analysis. **A:** A Manhattan plot displaying the MLMA performed in GCTA. The association analysis was based on 94,697 SNP markers and 731 dogs (324 cases and 407 controls). The significance level (blue line) was set to 1.24 × 10^–05^ using Bonferroni correction to adjust for multiple testing considering the LD, haploblock structure and number of independent SNPs after pruning the data. A second significance level (red line) was set to 5.28 × 10^–07^, using Bonferroni correction to adjust for all markers in the data. **B:** A quantile–quantile (q-q) plot showing the expected *p* value against the observed *p*-values of the MLMA

**Fig. 2:**
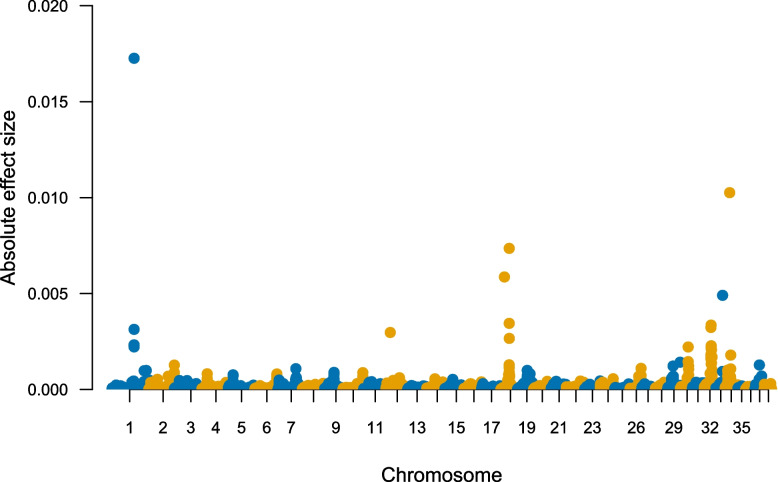
A Manhattan plot of the absolute SNP effect estimated in BayesR over the 38 autosomal chromosomes. The analysis was based on 97,185 SNP markers and 731 dogs (324 cases and 407 controls)

**Fig. 3:**
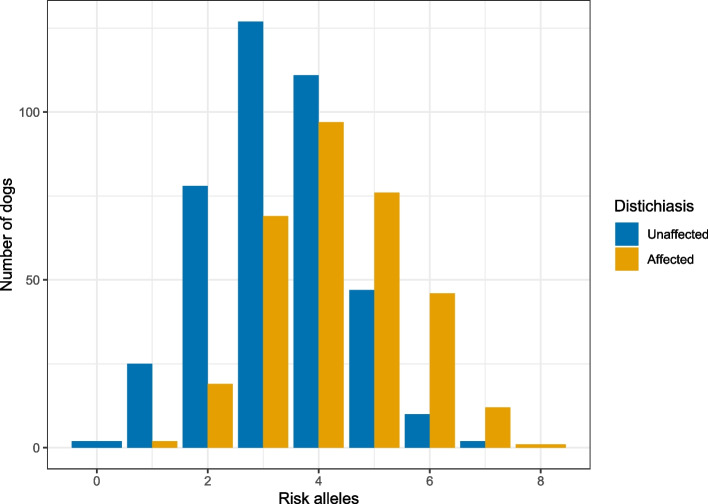
The total load of the risk alleles in the four loci identified in the MLMA performed in GCTA, including 324 cases and 407 controls

The association study was based on 731 SBTs (407 controls and 324 cases), and 94,697 autosomal markers. Four genomic regions were identified using a mixed linear model-based association analysis (MLMA) in Genome-wide Complex Trait Analysis (GCTA) [27], located on CFA1, CFA18, CFA32 and CFA34 (Fig. 1, Table 1). The same genomic regions on CFA1, CFA18 and CFA34 obtained an absolute effect size above 0.005 in BayesR. However, the SNP on CFA32 received a lower signal in the Bayesian model (Fig. 2, the posterior probability is displayed in Supplementary Fig. 1). The total load of the top four risk alleles in cases and controls is presented in Fig. 3. There is a significant difference in the total risk allele load between cases and controls (*p* < 2.0 × 10^–16^). On average, affected dogs carry 4.3 risk alleles and unaffected 3.3. risk alleles.

**Table 1:** The top four associated SNPs

Chr	SNP	BP position	Risk allele /Protective allele	Risk allele frequency affected	Risk allele frequency unaffected	OR	95% CI	*P*-value (MLMA)	Absolute effect size (BayesR)
1	BICF2P714726	73,777,342	G /A	0.94	0.85	2.66	1.84—3.85	1.90 × 10^–06^	0.017
18	BICF2P1386405	27,949,474	T/C	0.34	0.20	2.03	1.60—2.57	9.42 × 10^–06^	0.007
32	BICF2G630590287	17,605,832	T/C	0.33	0.21	1.84	1.46—2.33	1.21 × 10^–05^	0.003
34	BICF2S232639	14,960,862	G/A	0.54	0.38	1.92	1.55—2.36	6.77 × 10^–06^	0.010

The four top SNPs identified in the association analysis in the MLMA in GCTA, and with the effect size from BayesR. The base pair position is given in can.fam4 reference genome. The association study was based on 731 phenotyped SBTs (407 controls and 324 cases), and 94,697 autosomal SNP markers

The SNP on CFA1, BICF2P714726 attains the strongest effect size in BayesR. It is situated in an intergenic region, flanked by the genes *GAS1* ~ 260 kb downstream and *TUT7* ~ 210 kb upstream for the SNP (haploblock structure within the region is displayed in Supplementary Fig. 2).

The top SNP on CFA18, BICF2P1386405, is also situated in an intergenic region, the closest gene is *LRRC4C* 123 kb upstream, and the next gene, *API5* is situated ~ 1.2 MP downstream. The top SNP lies in a haploblock with five other SNPs spanning a distance of 134 kb. All six SNPs within the haploblock lie in the same intergenic region (Supplementary Fig. 3).

BICF2G630590287 on CFA32 obtained the lowest signals in the MLMA and was less distinct in BayesR. The top SNP is situated in an intron of the *EMCN* gene (Supplementary Fig. 4).

The SNP on CFA34, BICF2S232639, reached the second highest signal and is situated in an intron in the *TPK1* gene, and lies within a haploblock with nine adjacent SNPs spanning a distance of 850 kb (Supplementary Fig. 5). Other genes within the haploblock are *SOX2* 65 kb (upstream) and *DNJAC* (583 kb), and *FXR1* (582 kb) (both downstream).

### Population structure and relationships

Visual inspection of the principal components analysis (PCA) plots showed no general stratification. PCA plots including country of origin, genotyping arrays and cases and controls, are included in Supplementary Figs. 6 and 7. According to the registration number, the genotyped dogs are mainly Norwegian, followed by dogs from Sweden, reflecting the true population. The mean heterozygosity rate among the dogs in the dataset was 0.35. To assess some of the family structures in the dataset, 79 dogs that had an equal affection status as another sibling were excluded from an additional analysis (Supplementary Fig. 8). The SNP on CFA1 and CFA18 had a reduced significance compared with the analysis, including all dogs, while the SNPs on CFA32 and CFA34 had slightly higher significance. The top SNPs in the regions remained constant. The minor allele frequency of the four top SNPs between the two arrays was found to be at a similar level (Supplementary Table 1).

### Prediction of genomic values

Genomic values of the dogs were predicted using GBLUP in GCTA and BayesR when their phenotypes were masked in the six-fold cross-validation design. The two methods were compared by calculating the area under the curve (AUC) from a receiver operating characteristic curve (ROC curve). There was no significant difference (*P* = 0.984) in the AUC between the two methods. The AUC was 0.655 (CI 0.612–0.699) in GBLUP (Supplementary Fig. 9) and 0.651 (CI 0.607–0.695) in BayesR (Supplementary Fig. 10). Both methods gave a significant difference between the predicted genetic value (GV) in cases and control *P* = 4.12 × 10^–10^ in GBLUP and *P* = 3.56 × 10^–08^ using BayesR. Comparing the odds of the 25% of dogs with the highest GV estimate (most likely to develop distichiasis), with the 25% of dogs with a GV least likely to develop the disease, the odds ratio was 4.02 (95% CI 2.48–6.63) in GBLUP and 3.86 (95% CI 2.31- 6.55) in BayesR. No covariates had any effect on the performance of the models (Supplementary Table 2).

## Discussion

We have identified four potential candidate regions on CFA1, 18, 32 and 34 associated with distichiasis. The four risk alleles represent four novel genomic regions associated with distichiasis. *CTNND1,* seen in connection with "Blepharocheilodontic syndrome" with distichiasis in humans [20], is situated on CFA18 but more than 10 MB upstream from the top SNP on CFA18. The genetic mechanisms for developing distichiasis in humans, bovines and Friesian horses appear different from those in SBTs since none of the identified loci in these species overlaps with the loci identified in this study. The diversity of genes and genetic regions associated with distichiasis implies a significant genetic heterogeneity, where multiple loci lead to similar phenotypes.

There are several genes of interest within the associated genomic regions. *GAS1,* located around 260 kb downstream of the top SNP on *CFA1*, is involved in growth suppression, apoptosis and embryonal development [28]. *SOX2* is located 65 kb away from the top SNP on CFA34. *SOX2* is a transcription factor involved in regulating embryonic development [29]. On CFA18, *API5* is the closest gene to the top SNP. *API5* is an apoptosis inhibitor [30].

A GWAS intends to detect markers in LD with the causal variant. The LD within a single dog breed can be extensive and span regions of several megabases [31, 32]. This makes it challenging to pinpoint the actual causal variant. Adding related dog breeds could break up stretches of LD and might help identify causal variants.

We have set the significance level at 1.24 × 10^–05^, according to the number of independent SNPs after pruning the data, and after considering the LD structure and haploblock sizes. This is the same levels as suggested by Karlsson et al. based on the average size of 1 MB of independent haploblocks in a 2.4 GB dog genome [33], and the significase level sugested by Hayward et al. within breeds [34]. This significance level is, however, less stringent than a Bonferroni correction based on the number of all 94,697 markers in the dataset, which assumes all these markers are independent. When using a Bonferroni correction, none of the four genomic regions reaches significance.

The dataset contained genotyped dogs from two Illumina arrays, only the SNPs shared between the two arrays were used. Potential batch effects were assessed during the quality control. Including batch effect as a covariate did not have any effect on the genomic prediction. There is a high level of relationship between the dogs in the dataset. The GRM included in the mixed linear model account for part of this relationship. However, keeping siblings from the same litter may introduce some bias due to shared environment in early life and maternal effects. To account for such effects, we ran one analysis after removing siblings of equal affection status; however, the same four top SNPs remained.

In our study, the four genomic regions disclosed contribute only with a moderate effect on the phenotype. The top SNP on CAF32 obtained the lowest significance among the top SNPs in the MLMA and received low signals in BayesR. At the top SNP on CFA1, the minor allele (A) is protective, and carrying the major risk allele, G, gave 2.66 increased odds of developing distichiasis. In the other three SNPs, the minor allele is the risk allele and carrying one of the risk alleles gives a twofold increased risk of developing distichiasis. The chance of developing distichiasis increased with the number of risk alleles.

In a previous study conducted on the same SBT population, we found that most SBTs was only mildly affected by distichiasis [6]. Additionally, it has been observed that single *distichia* may be difficult to observe [2, 35]. As a result, some false negative controls are expected. However, the previously estimated heritability from a subset of the same SBT population using both SNP data and pedigree data was between ~ 0.37 and ~ 0.48 [6]. These estimates are consistent with other findings in the literature, which indicates that any recording errors are not so substantial that they reduce the genetic versus error variance ratio.

According to the registration in The Norwegian Kennel Club (NKK), the prevalence of distichiasis in the SBTs has persisted over the last twenty years [5]. Marker-assisted DNA- testing could help identify dogs with an increased risk of carrying disease alleles and identify the best dogs for breeding. The use of DNA-based risk tests for complex traits is challenging due to multiple casual loci with varying effect sizes. Additionally, the predictive value may differ between distinct populations due to differences in LD between the markers and the causal loci.

In complex traits where the effect size of most risk alleles is small and therefore not captured by the association analysis, genomic prediction, including the combined effect of all SNP markers across the whole genome, can be used to predict phenotypes [36]. Since breeding populations in dogs are often small compared to humans and livestock, and there are few examples of genomic prediction in dogs, our intention was to compare the two methods, BayesR and GBLUP, to predict genomic values for distichiasis in SBTs. BayesR has been shown to give more accurate predictions in human disease traits with loci of large effects compared with traditional mixed models [37]. The two approaches, GBLUP and BayesR, performed similarly within our dataset, which may be because our dataset was too small to accurately distinguish non-causal loci from causal loci with moderate to small effects.

There was a significant difference between the GV predicted from all 95 K SNPs between the cases and controls. Comparing the 25% of dogs most likely to acquire distichiasis with the 25% of dogs with the "best" GV (least likely to develop the disease) gave four times increased risk of developing distichiasis in the first group. The predictive accuracy for the individual dog was low and can, therefore, not be used to predict the phenotype in individual dogs. However, using the GVs at the breed level in the same manner as traditional pedigree-based breeding values, it should be possible to reduce the prevalence of distichiasis in the SBT population. Even if, on average, there would be an improvement in the population, the number of dogs with a "false" prediction may represent a challenge for the communication with the breeders.

Prediction of complex traits using genomic data typically requires large training datasets and testing in independent data sets [38–40], which may be challenging in small dog breeds. Edwards et al. [41] demonstrated that combining genomic predictions from two dog populations from different countries, even within the same breed, can reduce the prediction accuracy. This may be due to differences in LD between the two populations, different genes being important for the disease in the populations and recording differences. There is, therefore, a great need to evaluate the benefit of genomic selection in dog populations, and how to combine data across populations.

The material includes only dogs with a phenotype and represents only a subset of the overall SBT population. However, because most dogs used for breeding undergoes an eye examination, we believe the material is representative of the breeding population.

The use of imported SBTs in breeding is extensive in Norway, and more than half of the litters are from combinations where at least one parent is registered in another country. This can increase the genetic variation within the population and reduce the accuracy of genomic prediction.

Thorsrud et al. reported a higher AUC (0.94 with GBLUP) in their genomic prediction of distichiasis compared to our AUC of 0.66 with GPLUP. The divergent results between the SBT and guide dog populations emphasize that the results from genomic predictions of one disease trait may not be easily transferable between different breeds or populations and depend on the heritability and genetic complexity of the disease, number of disease cases and controls, and population structure and effective population size.

## Conclusion

Our study indicates that distichiasis in SBT is a complex trait with multiple genetic loci involved. We have identified four potential genomic regions on CFA1, 18, 32 and 34. Further studies must be conducted to validate the findings.

The genomic prediction, estimated from the joint effect of all 94,697 SNP, has the potential to aid in selective breeding, to reduce the prevalence of distichiasis in the SBTs but has a low predictive value for phenotypes in individual dogs. The genomic prediction of distichiasis must be validated in each other target population.

## Material and methods

A subset of SBTs with an official eye examination record registered by NKK between 2005 and April 2022 were included. Eye examinations were performed by veterinarians certified by the European College of Veterinary Ophthalmologists (ECVO). The eye examination records are available in "dogweb", an open database established by NKK (www.dogweb.no). The dogs were classified as affected or unaffected according to the diagnosis on the eye examination records. In our study, dogs with a positive distichiasis diagnosis were regarded as affected (case) regardless of examination age and a later negative examination. Dogs were considered unaffected (controls) when diagnosed as negative for distichiasis after one year of age. This is consistent with the findings from a previous study where we found that a negative distichiasis status in puppies did not give a reliable picture of the distichiasis status in the adult dog [6].

Samples were collected from a biobank established in collaboration between the Norwegian University of Life Sciences (NMBU) and the NKK. DNA from EDTA blood was extracted using E.Z.N.A. Blood DNA Mini Kit from Omega, following the manufacturer's description. The DNA quality was measured with Epoch from BioTek. Seven hundred and thirty-four samples were genotyped on the Illumina 220 K CanineHD Bead chip (Neogen Genomics, USA), and 118 samples from a previous study were genotyped on the Illumina 170 K CanineHD bead chip. Only the 170 K markers shared between the two datasets were kept in the joint analysis.

### Quality control

Quality control was performed in Plink 1.9 [42, 43] and in R, using base R and the R package Tidyverse [44, 45]. At the individual level, we removed samples with a genotyping rate below 95% and a heterozygosity rate above three standard deviations from the mean. We controlled for sex mismatch to identify potential sample mix-ups and removed duplicates. At the marker level, we eliminated markers with a call rate below 98%, a minor allele frequency below 0.05, and deviation from Hardy–Weinberg equilibrium at a level of -1.0 × 10^–6^ in controls and -1.0 × 10^–10^ in cases, using the Fisher exact test incorporated in Plink. Dogs with a missing phenotype were removed. To assess potential batch effects of the two arrays 170 K and 220 K, a PCA plot was constructed. In addition, the SNP markers were regressed on the two batch (170 K and 220 K) to assess differences in the allele frequency in the two batches. After quality control, the material consisted of 97,185 markers and 731 dogs, where 407 were controls and 324 cases, 442 female (206 cases) and 289 males (118 cases); Seventy-six dogs (16 cases) were genotyped on the 170 k array, and 655 dogs (308 cases) were genotyped on the 220 k array. Mean age of last eye examination in the controls was 2.7 years and 1.9 years in the cases.

### Population structure and LD

There is no data on the current population size of SBT in Norway. According to NKK there is around 1000 new registrations of SBTs every year. Between 2005 and April 2022, NKK had registered 1481 imported dogs from 30 different countries, the majority imported from Sweden (52,65%), followed by the United Kingdom (10.05%). Among 2339 litters registered during the same time period, 1493 (61.52%) litters were of mattings with at least one parent from another country. Population structure attributable to country of origin (according to pedigree number), was assessed using principal components analysis conducted in Plink. Plots were constructed in R using the R package ggplot2 [46]. In addition, population structure due to stratification between cases and controls was assessed.

The dataset contained 72 families with offspring, and both parents genotyped with an equal number of affected and unaffected offspring. The total number of genotyped full siblings was 220, distributed on 96 different litters. A GWAS excluding 79 dogs with an equal affection status as another litter mate was conducted.

Linkage disequilibrium (LD) and haploblock size were estimated using Plink 1.9 [42, 43] and R [44]. Haploblock sizes were estimated using the *–blocks* function. Plink uses the haploblock definition suggested by Gabriel et al. [47]. To identify markers in pairwise LD, and to estimate the number of independent SNP markers, we used the LD pruning function in Plink: *indep-pairwise* with the options; window size: 50, step size: 5 and r^2^: 0.2. The mean heterozygosity rate was estimated in Plink using the *–het* function.

### Association analysis

The association analysis was performed in BayesR (v01/04/2021) [37]. BayesR fit all markers simultaneously, and there are indications that the Bayesian model has a higher power to detect true associations and SNP effect than traditional linear models. In addition, BayesR gives information about the genetic architecture of the trait. BayesR uses the model:

$$y={1}_{n}\mu +Xa+e$$

where *y* = a vector of the phenotypes, *μ* is the general mean term, *X* is a matrix of the genotypes, and *a* a vector of SNP effects, and *e* is a vector of residual errors [37]. BayesR uses a prior of four predefined classes of SNP effects, with the normal distributions *N*($\mathrm{0,0}*{\sigma }_{g}^{2}$), $N(\mathrm{0,0.0001}*{\sigma }_{g}^{2})$, $N(\mathrm{0,0.001}*{\sigma }_{g}^{2})$ and $N(\mathrm{0,0.01}*{\sigma }_{g)}^{2}$. The variance of the SNP effect (${\sigma }_{g}^{2}$) is defined by the data, using a Gibbs sampler to draw samples. Our analysis was run with 100.000 iterations and 50.000 burn-in steps.

In addition, a traditional mixed linear model-based association analysis (MLMA) was run in Genome-wide Complex Trait Analysis (GCTA) version 1.93.2 [27, 48]. A relationship matrix (GRM) is used to control for population structure and relationships.

$$y=\alpha +\beta X+g+e$$

where y = a vector of the phenotype, α is the general mean term, *β* the fixed additive genetic effect of the SNP considered in the analysis, *X* = genotype of the SNP coded as 0, 1 and 2 for homozygous, heterozygous and opposite homozygous, respectively, *g is* the random effect of background genes assumed distributed as *g* ~ N(0,GRM), and *e* is the residual error. We have used two significance levels, one using Bonferroni correction according to number of all SNPs in the dataset (0.05/94697 = 5.28 × 10^–07^). Bonferroni correction based on all SNP markers is often considered over-conservative because SNPs are in LD and not independent. Therefore, we calculated a second significance level in accordance with the number of independent SNPs after pruning the data (0.05/4032 = 1.24 × 10^–05^). Manhattan plots were made in R with the qqman package [49].

The genomic position on the arrays was given in CanFam3.1 [50]. To convert the genomic positions from CanFam3.1 to GSD_1.0 /canFam4 reference genome [51], we used the Liftover tool developed by the University of California Santa Cruz Genomes [52]. All genomic positions refer to GSD_1.0 /canFam4.

### Candidate regions

A t-test in R was used to assess if there was a significant difference in the mean risk allele load between cases and controls [44]. Haploblocks within the four candidate regions were analyzed and visualized using Haploview [53].

### Prediction

Two approaches were used to predict the dogs' genetic value (GV) based on whole genome SNP markers. Genomic best linear unbiased prediction (GBLUP) calculated in GCTA [27] and a Bayesian hierarchical model, BayesR [37, 54]. GBLUP in GCTA assume a normal distribution of the SNP effect and is based on a mixed linear model:

$$y=\alpha +g+ e$$

where *y* is a vector of the phenotypes, α the mean term, *g* is the genetic value and *ε* the residual error. The values of* g* and *e* were estimated from the formulas: *ĝ* = *V*_*g*_*AV*^*−1*^*y* and *ê* = *V*_*e*_*V*^*−1*^*y, wh*ere A is the GRM, *Vg* the genetic variance and *Ve* residual variance and V = A*V_g_ + I*V_e_ is the variance matrix of the records y [27, 48]. The prediction in BayesR is based on the same mixed models as described in the association analysis. The same mixture of four normal distributions of SNP effects was applied for the prediction. For the prediction, 20.000 burn-in steps and 50.000 iterations were used. Full siblings were removed from the dataset prior to the prediction. The dataset consisted of 94,697 markers across the 38 autosomal chromosomes and 607 dogs, including 248 cases and 359 controls. A sixfold cross-validation was used. To assess the two methods' ability to discriminate between cases and controls, we used the R package pROC [55] to compute the AUC from the ROC curves, with sensitivity on the y-axis and specificity on the x-axis. Different models were tested in GBLUP, GCTA; the base model with no covariables, and models including the covariables: sex, examination age, batch effect, ten first PCA and country of origin. None of the tested covariables improved the model. Therefore, in the final estimates, the base model with no covariates was used. Delong's test in pROC was used to detect a significant difference between the two approaches, BayesR and GBLUP in GCTA.

We compared the odds of developing distichiasis in the 25% of dogs with GV predicted to be most likely to develop the disease, with the odds of developing distichiasis of the 25% of dogs with GV least likely to acquire distichiasis.

## Supplementary Information

**Additional file 1.**

## Data Availability

The genomic data are available from the corresponding author upon reasonable request. All phenotype data are available from the open database "Dogweb", www.dogweb.no.
