# Supplementary material for: Genomic analysis and prediction of genomic values for distichiasis in Staffordshire bull terriers
Source: Canine Med Genet. 2023 Jul 24;10:9. doi: 10.1186/s40575-023-00132-1 (PMC10367371; doi:10.1186/s40575-023-00132-1)
Supplement: Supplementary file 1 — Additional file 1. [file 40575_2023_132_MOESM1_ESM.docx]

# Additional material


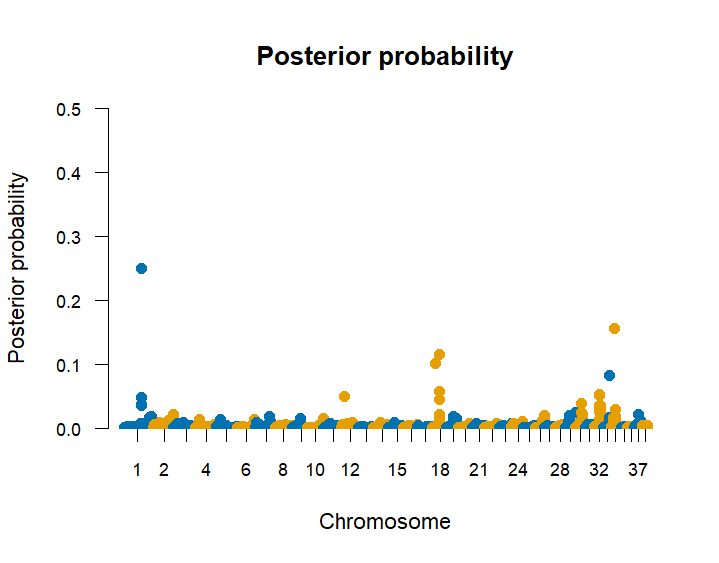


**Supplementary Figure 1**. The posterior inclusion probabilities of the SNP in the fourth mixture class (N(0,0.01*σ_(g))^2) from the analysis conducted in BayesR.


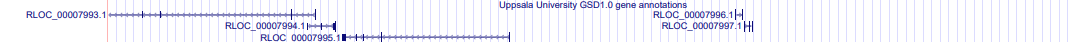

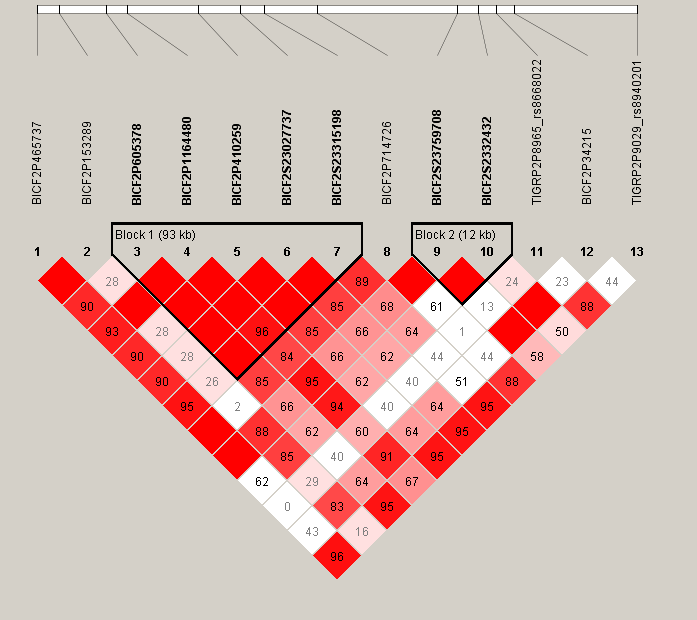


**Supplementary Figure 2**. Haploblock from the candidate region on chr 1. shows the top SNP, BICF2P714726 situated between two haploblocks. The numbers in the squares show the level of LD, based on the D prime (D’). The colour shadings are based on the Lod score and D’ estimated between two SNPs. A D' < 1 and LOD < 2 gives a white colour, D' < 1 and LOD ≥ 2 gives shades of pink. D' = 1 and LOD < 2 give blue colour. D' = 1 and LOD ≥ 2 give dark bright red colour. (<https://www.broadinstitute.org/haploview/ld-display>). On the top of the figure is a picture of the corresponding genomic region from UCSCs genome browser (<https://genome.ucsc.edu>), with the position from canFam4 reference genome.


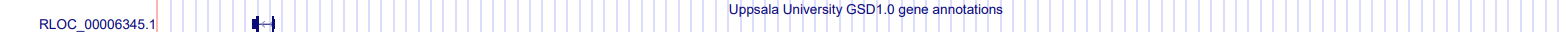

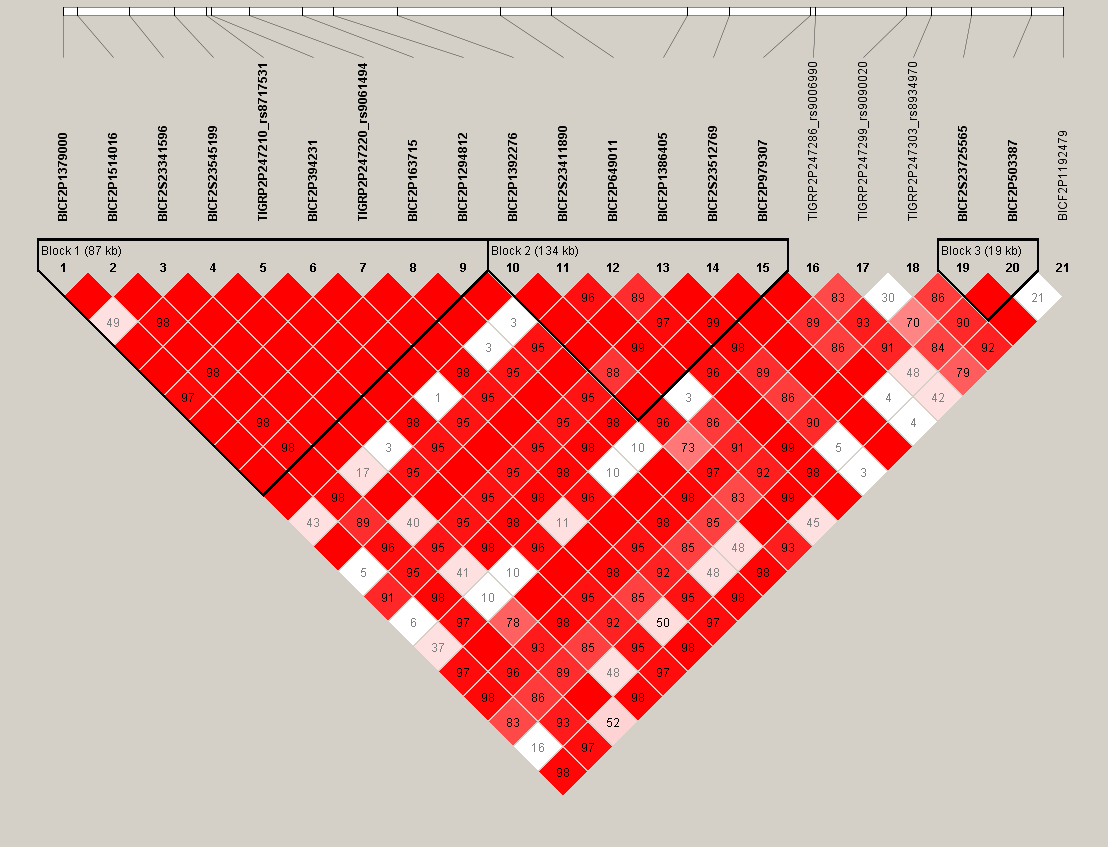


**Supplementary Figure 3**. Haploblocks in the candidate region on chr 18, the top SNP BICF2P1386405 is situated in the second haploblock with five other SNPs. The numbers in the squares show the level of LD, based on the D prime (D’). The colour shadings are based on the Lod score and D’ estimated between two SNPs. A D' < 1 and LOD < 2 gives a white colour, D' < 1 and LOD ≥ 2 gives shades of pink. D' = 1 and LOD < 2 give blue colour. D' = 1 and LOD ≥ 2 give dark bright red colour. (<https://www.broadinstitute.org/haploview/ld-display>). On the top of the figure is a picture of the corresponding genomic region from UCSCs genome browser (<https://genome.ucsc.edu>), with the position from canFam4 reference genome.


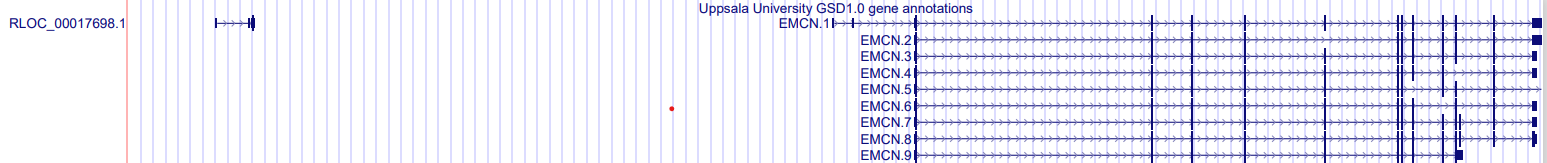

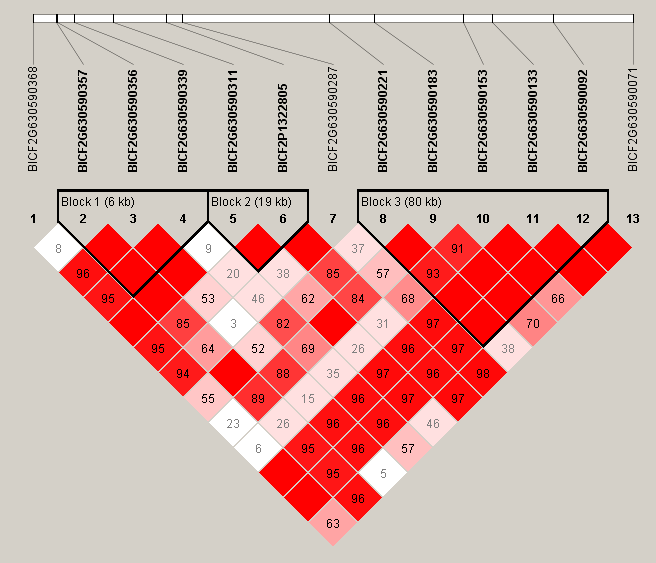


**Supplementary Figure 4**. Haploblocks chr 32, the top SNP BICF2G630590287 lays between haploblock 2 and 3. The numbers in the squares show the level of LD, based on the D prime (D’). The color shadings are based on the Lod score and D’ estimated between two SNPs. A D' < 1 and LOD < 2 gives a white colour, D' < 1 and LOD ≥ 2 gives shades of pink. D' = 1 and LOD < 2 give blue colour. D' = 1 and LOD ≥ 2 give dark bright red colour. (<https://www.broadinstitute.org/haploview/ld-display>). On the top of the figure is a picture of the corresponding genomic region from UCSCs genome browser (<https://genome.ucsc.edu>), with the position from canFam4 reference genome.


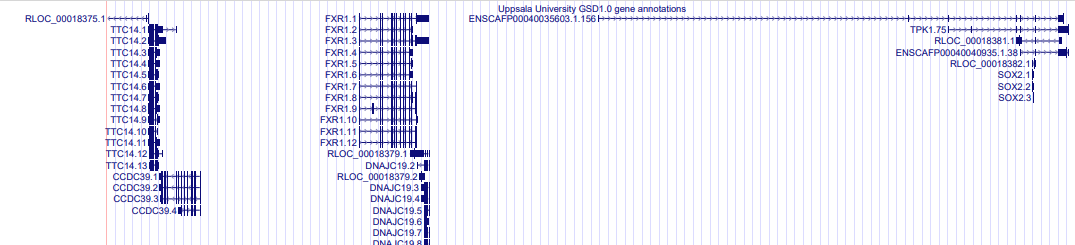


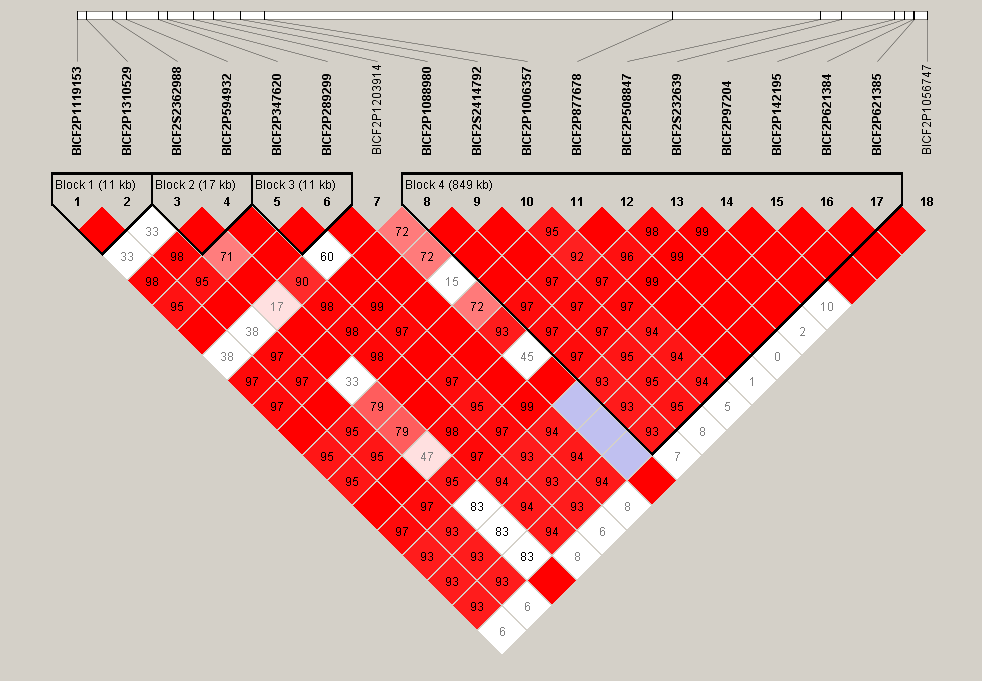


**Supplementary Figure 5**. Haploblocks chr 34, the top SNP BICF2S232639 is situated in the middle of haploblock 4 with nine other SNPs. The numbers in the squares show the level of LD, based on the D prime (D’). The colour shadings are based on the Lod score and D’ estimated between two SNPs. A D' < 1 and LOD < 2 gives a white colour, D' < 1 and LOD ≥ 2 gives shades of pink. D' = 1 and LOD < 2 give blue colour. D' = 1 and LOD ≥ 2 give dark bright red colour. (<https://www.broadinstitute.org/haploview/ld-display>). On the top of the figure is a picture of the corresponding genomic region from UCSCs genome browser (https://genome.ucsc.edu), with the positions from canFam4 reference genome.


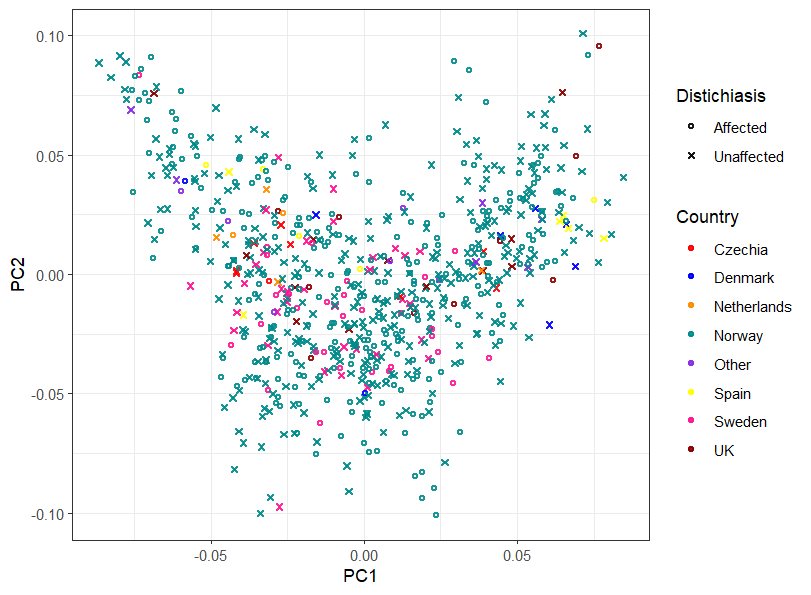


**Supplementary Figure 6.** PCA plot, including the country of origin. Other countries are countries with less than five genotyped dogs. This includes dogs from Finland, Germany, Greece, Poland, Malta, Austria, and Hungary.


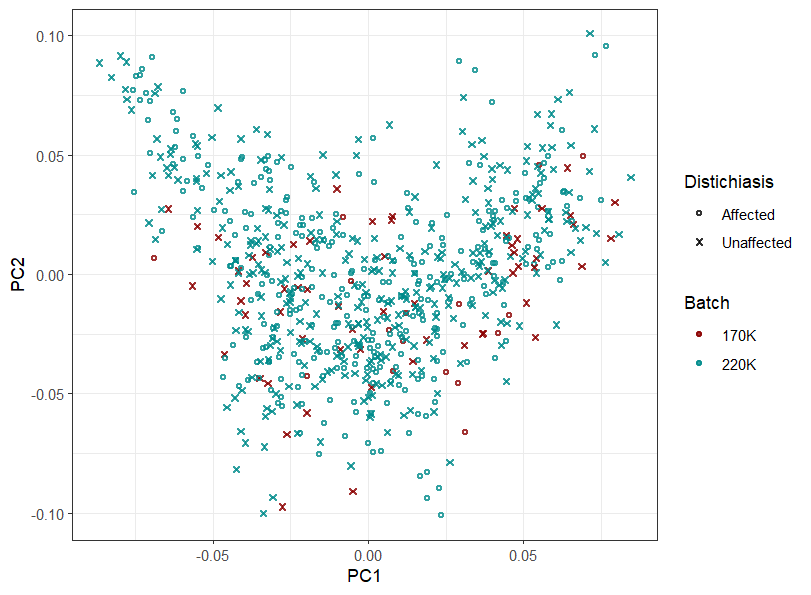


**Supplementary Figure 7**. A PCA plot showing the distribution of affected and unaffected dogs in the two arrays, 170K and 220K.


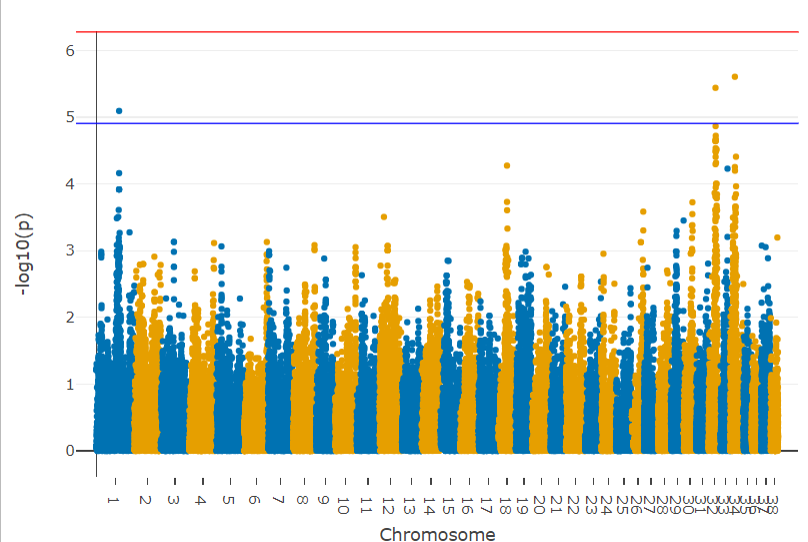


***Supplementary Figure 8.*** *A Manhattan plot where 79 siblings with an equal affection status as full siblings are removed. Includes 652 dogs, 377 are controls, and 275 are cases. The significance level (represented by the blue line) is set to 1.24 x 10^-05^ using a Bonferroni correction to account for multiple testing using the number of independent markers (4030), a second significance level of 5.28 x 10^-07^ (represented by the red line) is the Bonferroni correction based on all markers 94697.*

**Supplementary Table 1**. Minor allele frequency (MAF) of the four top SNPs on the two arrays.

| CHR | SNP | MAF 170K Array | MAF 220K Array |
| --- | --- | --- | --- |
| 1 | BICF2P714726 | 0.12 | 0.11 |
| 18 | BICF2P1386405 | 0.20 | 0.27 |
| 32 | BICF2G630590287 | 0.24 | 0.26 |
| 34 | BICF2S232639 | 0.43 | 0.45 |

The 170K array includes 76 dogs (16 cases and 60 controls), and the 220K array includes 655 dogs (308 cases and 347 controls).


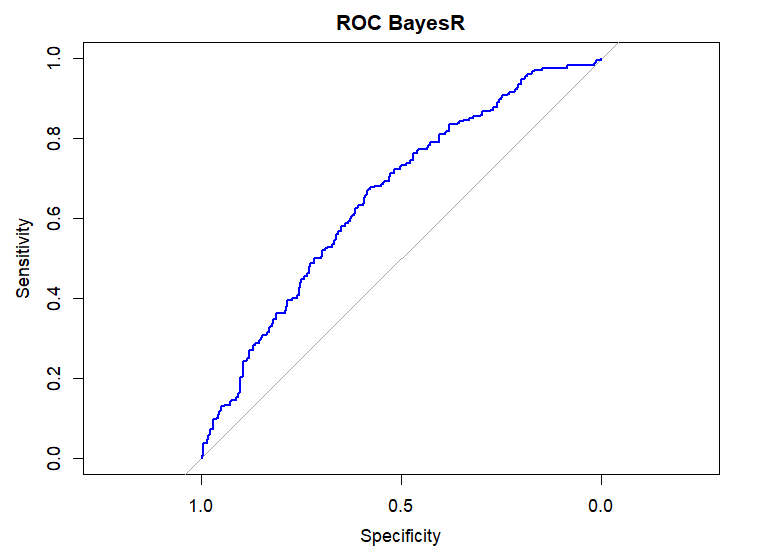

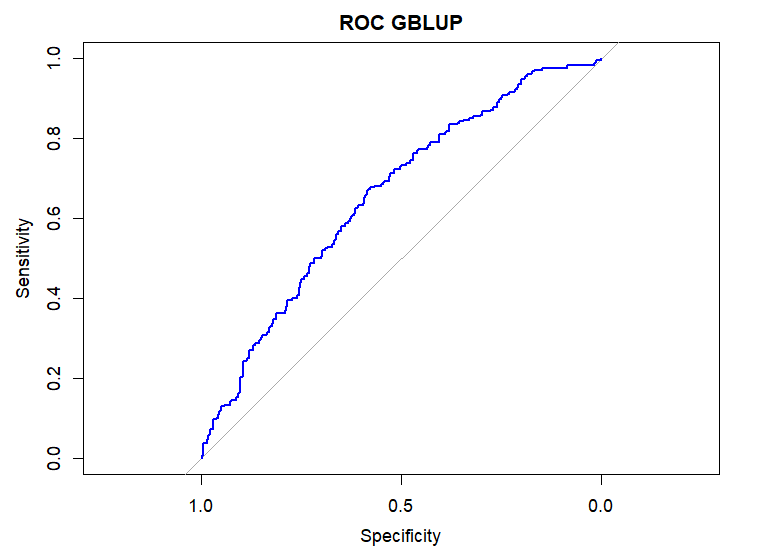


**Supplementary Figure 9**. A ROC curve calculated from the estimated GV using GBLUP.

**Supplementary Figure 10**. A ROC curve calculated from the estimated GV using BayesR.

**Supplementary Table 2**. 6-fold cross validation in GBLUP including covariables

| Covariable | AUC | Confidence interval |
| --- | --- | --- |
| Sex | 0.66 | 0.61-0.70 |
| Batch | 0.65 | 0.61-0.69 |
| Country of origin | 0.66 | 0.61-0.70 |
| Age | 0.65 | 0.60-0.69 |
| PCA | 0.64 | 0.60-0.69 |

*The area under the curve (AUC) calculated from the receiver operating characteristic curve (ROC curve) from all estimated genetic values in the GBLUP. Batch indicates the 170K and 220K array, country of origin according to the registration number, and age is the examination age. PCA includes the first teen first principal components analysis (PCA).*
